# Shooting for the Moon: Can We Cut Cancer Mortality in Canada By 50% By 2050?

**DOI:** 10.1177/10732748251319485

**Published:** 2025-02-13

**Authors:** Keaton Banik, Yibing Ruan, Mariet M. Stephen, John M. Hutchinson, Chantelle Carbonell, Matthew T. Warkentin, Andrew Coldman, Rochelle Garner, Hawre Jalal, Darren R. Brenner

**Affiliations:** 1 Department of Oncology, Cumming School of Medicine, 213572 University of Calgary, Calgary, AB, Canada; 2 Department of Cancer Epidemiology and Prevention Research, Cancer Care Alberta, Alberta Health Services, Calgary, AB, Canada; 3 8162 British Columbia Cancer Research Institute, Vancouver, BC, Canada; 4 6360 Health Analysis Division, Statistics Canada, Ottawa, ON, Canada; 5 School of Epidemiology and Public Health, 177403 University of Ottawa, Ottawa, ON, Canada; 6 Department of Community Health Sciences, Cumming School of Medicine, 213572 University of Calgary, Calgary, AB, Canada

**Keywords:** cancer epidemiology, prevention, screening, cancer mortality, microsimulation

## Abstract

**Introduction:**

The United States of America reignited their Cancer Moonshot Initiative in 2022 with an ambitious goal to reduce cancer mortality by 50% over the next 25 years. In this study, we estimated how and whether a similar cancer control initiative could be achieved in Canada.

**Methods:**

We used the OncoSim microsimulation suite to address three questions: (1) what is the expected mortality from cancer in Canada by 2050 given the current trends?; (2) what would be the maximal impact on reducing cancer mortality with prevention and increased screening activities? and, (3) if a 50% reduction in projected cancer mortality could not be achieved through the primary and secondary intervention efforts, what additional advancements and discoveries would be needed to fill the “lunar gap”? We modeled the joint impact of risk-factor reduction and screening, as well as the independent effects of prevention and screening alone, on projected cancer mortality.

**Results:**

Our models suggest that there will be an expected 133,395 cancer deaths in 2050 in Canada. Approximately 33% of these cancer deaths could be prevented by risk-factor reduction and increased screening programs by the year 2050. This would leave a “lunar gap” of about 16%–17% that would need to be bridged with novel discoveries in cancer risk prevention, early detection, and treatment.

**Conclusion:**

While current knowledge and implementation of prevention and screening would have a considerable impact on a Canadian cancer moonshot, additional efforts are needed to implement cancer control initiatives and fuel additional discoveries to fill the gap.

## Introduction

In 2016, then Vice President Joe Biden announced the Cancer Moonshot initiative to reduce cancer mortality by 50% in the next 25 years and to improve the conditions of people living with or around cancer in the United States. Since its launch in 2017, $1.8 billion USD has been allocated to cancer research funding, leading to over 240 research projects and 70 programs.^1^ The Cancer Moonshot was relaunched in 2022 with a focus on accelerating cancer research.

In Canada, nearly half of the population is expected to be diagnosed with cancer in their lifetime, with a quarter of Canadians expected to die from cancer.^2^ Research suggests that over 30% of incident cancer cases in Canada can be attributed to modifiable and preventable risk factors.^3^ A reduction in these factors could prevent up to 40,000 cancer cases by 2042.^3^ The impact of a Cancer Moonshot in the United States raises questions around the potential of a similar initiative in Canada. Previous efforts have had a meaningful impact on avoiding excess cancer mortality in Canada.^4^ While a 50% decrease in cancer mortality is ambitious, the key to achieving this goal lies in multiple interventions across primary, secondary, and tertiary cancer prevention.

The microsimulation tool, OncoSim, can be used to project cancer outcomes that reflect observations in real-world population-level data in Canada.^5^ In this study, we simulated what would be required for a 50% reduction in the projected cancer mortality through a similar Cancer Moonshot in Canada. We aimed to answer three questions related to a potential moonshot program. First, based on the current trends, what is the projected cancer mortality in Canada by the year 2050? Second, what would be the impact on cancer mortality if the modifiable cancer risk factors could be reduced to minimum and the efficacy of the cancer screening programs could be maximized? Third, if a 50% reduction in projected cancer mortality could not be achieved through the aforementioned efforts, what level of impact would be needed through additional discoveries and advancements in cancer research to reach a 50% reduction in cancer mortality by 2050?

## Methods

Our goal was to estimate that, given the current decreasing trend of cancer mortality, what relative reduction in cancer mortality could be observed in 2050 if theoretically maximized primary and secondary preventions were achieved. Our estimations were based on simulations using the OncoSim microsimulation tool, which is developed and maintained by Statistics Canada and supported by the Canadian Partnership Against Cancer (CPAC).^6^ OncoSim has five models, including the All Cancers model, and the site-specific Breast, Lung, Colorectal and HPV/Cervical cancer models, which are tailored to estimate future cancer outcomes in response to changes in input parameters such as risk factor distribution, screening modality and performance, etc. These models have been used in a variety of studies to inform policies, compare screening modalities, or estimate the consequences of service interruptions.^6-12^ In OncoSim, the age and sex-specific cancer incidence and mortality rates are calibrated using data from the Canadian Cancer Registry with logistic regression analyses and one or two-piece Weibull distributions, respectively.^6^ Population growth was modeled with the medium-growth scenario from Statistics Canada, which take into accounts the population ageing.^13^ Therefore, the projected cancer incidence and deaths in OncoSim reflects the underlying trend and the ageing population. We estimated the impact of reducing the exposure of individual risk factors on cancer incidence and related mortality for all associated cancer sites. We also evaluated the effect of improving the performance of the existing screening programs for breast, cervical, colorectal, and lung cancer.

### Risk Factors

The OncoSim All Cancers model has 15 modifiable cancer-associated risk factors that can be used to estimate the effect of primary prevention. These include: active smoking, passive (i.e., secondhand) smoking among non-smokers, alcohol consumption, excess body fat (as measured by body mass index), inadequate physical activity, sedentary behavior, inadequate fruit intake, inadequate vegetable intake, red meat consumption, processed meat consumption, artificial UV radiation, air pollution (PM2.5), Hepatitis B virus, Hepatitis C virus, and *Helicobacter pylori*. The exposure prevalences were acquired from population-based surveys in Canada and the relative risks with associated cancers were obtained from high-quality meta-analyses, which have been used in the ComPARe study.^14^ We used the OncoSim-Lung model to estimate the effect of indoor radon mitigation on lung cancer mortality. We used the OncoSim-HPV/Cervical model to estimate the effect of HPV vaccination on cervical cancer mortality.

The counterfactual models for each risk factor imposed an intervention strategy to reduce its impact on cancer outcomes starting in 2025, resulting in each risk factor reaching minimal theoretical levels by 2030. The projected exposure level of the risk factors in 2025 and the theoretical minimal target level in 2030 are shown in Table 1. We assumed a 10-year latency period between the interventions and the effects on cancer incidence.^15^ Models projected cancer outcomes forward for the 20 years after the target risk-factor level was reached in 2030 (i.e., until the year 2050).

**Table 1.** Counterfactual Intervention Parameters for Each Modifiable Risk Factor Investigated in This Study.

Risk Factor	Exposure Level in 2025 (Projection of ComPARe)	Target Level in 2030	Note
Tobacco smoking	Female: Current 13.2%, former 39.4%, never 47.4%	Female: Current 0%, former 52.6%, never 47.4%	
	Male: Current 19.8%, former 46.9%, never 33.3%	Male: Current 0%, former 66.7%, never 33.3%	
Secondhand smoking	Female: 7.9%	Female: 0%	Exposure among non-smokers
	Male: 8.9%	Male: 0%	
Physical inactivity	Female: Inactive 38.9%, moderate 28.8%, active 32.3%	Female: Active 100%	
	Male: Inactive 37.6%, moderate 27.2%, active 35.1%	Male: Active 100%	
Leisure-time sedentary behaviour	Female: >6 hr/d 12.4%, 3-6 hr/d 79.2%, <3 hr/d 8.4%	Female: <3 hr/d 100%	
	Male: >6 hr/d 11.4%, 3-6 hr/d 78.8%, <3 hr/d 9.8%	Male: <3 hr/d 100%	
Body mass index	Female: Obese 22.0%, overweight 28.2%, normal 49.8%	Female: Normal 100%	
	Male: Obese 27.0%, overweight 36.4%, normal 36.6%	Male: Normal 100%	
Alcohol	Female: Nondrinker 5.6%, 0∼1 drink/day 76.8%, >1 drink/day 17.6%	Female: Nondrinker 100%	
	Male: Nondrinker 3.0%, 0∼1 drink/day 60.6%, >1 drink/day 36.4%	Male: Nondrinker 100%	
Insufficient vegetable intake	Female: <4 servings/d 66.5%, >=4 servings/d 33.5%	Female: >=4 servings/d 100%	
	Male: <4 servings/d 81.2%, >=4 servings/d 18.8%	Male: >=4 servings/d 100%	
Insufficient fruit intake	Female: <4 servings/d 86.4%, >=4 servings/d 13.6%	Female: >=4 servings/d 100%	
	Male: <4 servings/d 88.3%, >=4 servings/d 11.7%	Male: >=4 servings/d 100%	
Red meat	Female (age): [18,40) = 2.88 times/week, [40,60) = 2.66 times/week, [60,80) = 2.83 times/week	Female: zero times/week	Varies by age group
	Male (age): [18,40) = 3.98 times/week, [40,60) = 3.37 times/week, [60,80) = 3.16 times/week	Male: zero times/week	
Processed meat	Female (age): [18,40) = 1.13 times/week, [40,60) = 0.9 times/week, [60,80) = 0.86 times/week	Female: zero times/week	Varies by age group
	Male (age): [18,40) = 1.67 times/week, [40,60) = 1.23 times/week, [60,80) = 1.35 times/week	Male: zero times/week	
PM2.5	8.3 μg/m3	0.5 μg/m3	0.5 μg/m3 is the measurement of Zurich Switzerland, the city with the best air quality
Radon	Various level by house types and provinces	1 Bq/m3	1 Bq/m3 is the outdoor level radon
Artificial UV	Female (age): [16,25) = 58.3%, [25,44) = 50.7%, [44,∞) = 24.2%	Female: 0%	Varies by age group
	Male (age): [16,25) = 16.3%, [25,44) = 21.6%, [44,∞) = 11.8%	Male: 0%	
*Helicobacter pylori*	Female: <50yo 9.8% >=50yo 27.9%	Female: 0%	
	Male: <50yo 12.8% >=50yo 27.9%	Male: 0%	
HBV	Female (age): [18,30) = 0.32%, [30,40) = 0.70%, [40,50) = 0.39%, [50,60) = 0.15%, [60,70) = 0.35%, [70, ∞) = 0.12	Female: 0%	Varies by age group
	Male (age): [18,30) = 0.31%, [30,40) = 0.93%, [40,50) = 0.32%, [50,60) = 0.88%, [60,70) = 0.51%, [70, ∞) = 0.08	Male: 0%	
HCV	Female (age): [16,21) = 0.11%, [21,26) = 0.27%, [26,31) = 0.45%, [31,36) = 0.79%, [36,41) = 0.93%, [41,46) = 1.14%, [46,51) = 1.21%, [51,56) = 0.75%, [56,61) = 0.79, [61,66) = 0.89%, [66,71) = 0.70%, [71, ∞) = 0.47%	Female: 0%	Varies by age group
	Male (age): [16,21) = 0.20%, [21,26) = 0.48%, [26,31) = 0.76%, [31,36) = 1.29%, [36,41) = 1.50%, [41,46) = 1.82%, [46,51) = 1.88%, [51,56) = 1.05%, [56,61) = 1.04, [61,66) = 1.10%, [66,71) = 0.76%, [71, ∞) = 0.51%	Male: 0%	

In our model, the risk-factors prior to 2025 were set to the status quo level. Between the years 2025 and 2030, the counterfactual intervention exposure level was being adopted. Past year 2030, the full effect of the counterfactual intervention scenario was realized. Some risk-factors are age-dependent thus, multiple parameters are adjusted between the year 2025 and year 2030 exposure levels.

### Screening

Secondary cancer prevention consists of methods to detect cancers early or even precancerous stages among asymptomatic individuals in order to treat them with curative intent. To estimate avoidable cancer deaths through screening, we used the OncoSim-Breast, OncoSim-Lung, OncoSim-Colorectal, and OncoSim-HPV/Cervical model for these four cancers, which either have existing or pilot screening programs in Canada. We simulated a set of “status quo” scenarios, in which the screening eligibility and participation rates were held at current levels in Canada.^16-18^ In parallel, we simulated a set of scenarios with expanded eligibility (i.e., screening age), shorter screening interval (i.e., annual screening), and assumed 100% participation rates. For cervical cancer, we switched the screening modality from Pap tests to HPV DNA testing, which is proven to have higher sensitivity in detecting cancerous lesions.^19^ In these scenarios, risk-factor interventions (primary prevention) are left unchanged to remove additional bias when looking at the impact of secondary prevention (referred to as Screening-Only). The details of the parameter setup for the screening scenarios are presented in Supplemental Table 1.

### Combined Effect

To estimate the overall reduction in the mortality of individual cancer sites from the interventions of multiple risk factors, we applied the adjustment method for multiple risk factors as described in a previous study.^3^ In brief, for cancers associated with multiple risk factors that are not mutually exclusive in exposure, the combined fraction of cancer death that could be prevented is 100% minus the product of the non-attributable fraction of cancer death of each risk factor. To incorporate the effect of screening with risk factor intervention, we assumed that screening was only effective in detecting cancers that were not prevented through risk factor intervention. The net effect of screening was estimated as the proportion of cancer deaths prevented through screening multiplied with the proportion of cancer deaths not prevented through risk factor intervention. The combined effect of primary and secondary intervention was estimated as the sum of the proportion of deaths prevented through risk factor intervention and the net effect of screening. The remaining percentage required to reach a 50% reduction in cancer deaths was proposed to be the gap that needs to be filled by future advancements and tertiary prevention (treatment of individuals to prevent premature cancer mortality): we refer to this as the “lunar gap”. We also examined the effect of screening programs separately from risk-factor reduction (Screening-Only) when comparing results.

## Results

### Projected Cancer Mortality by 2050

**Figure 1.**
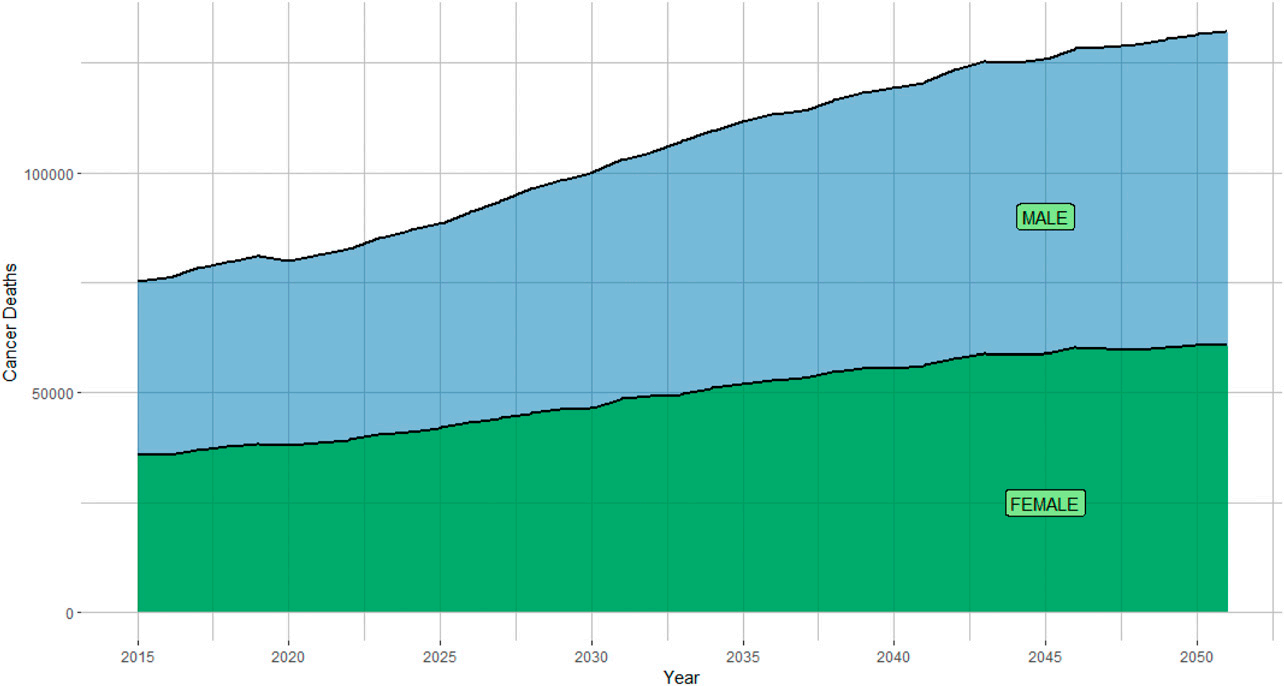
Status-quo projections of cancer deaths to the year 2050. The area under the curve is divided to show the ratio between male and female deaths.

Our status-quo model projected 133,398 total cancer deaths in the year 2050, 53% (70,736) of which would occur among males (Figure 1). Lung cancer was responsible for the most cancer mortality with a projected 19,745 deaths in males and 19,171 deaths in females in the year 2050. Colorectal and breast cancer deaths were the second and third most common for females, while colorectal and prostate cancer deaths were the second and third most common for males. Cancer deaths by each cancer type in our status-quo model are shown in Supplemental Table 2.

### Risk Factor Reduction

**Figure 2.**
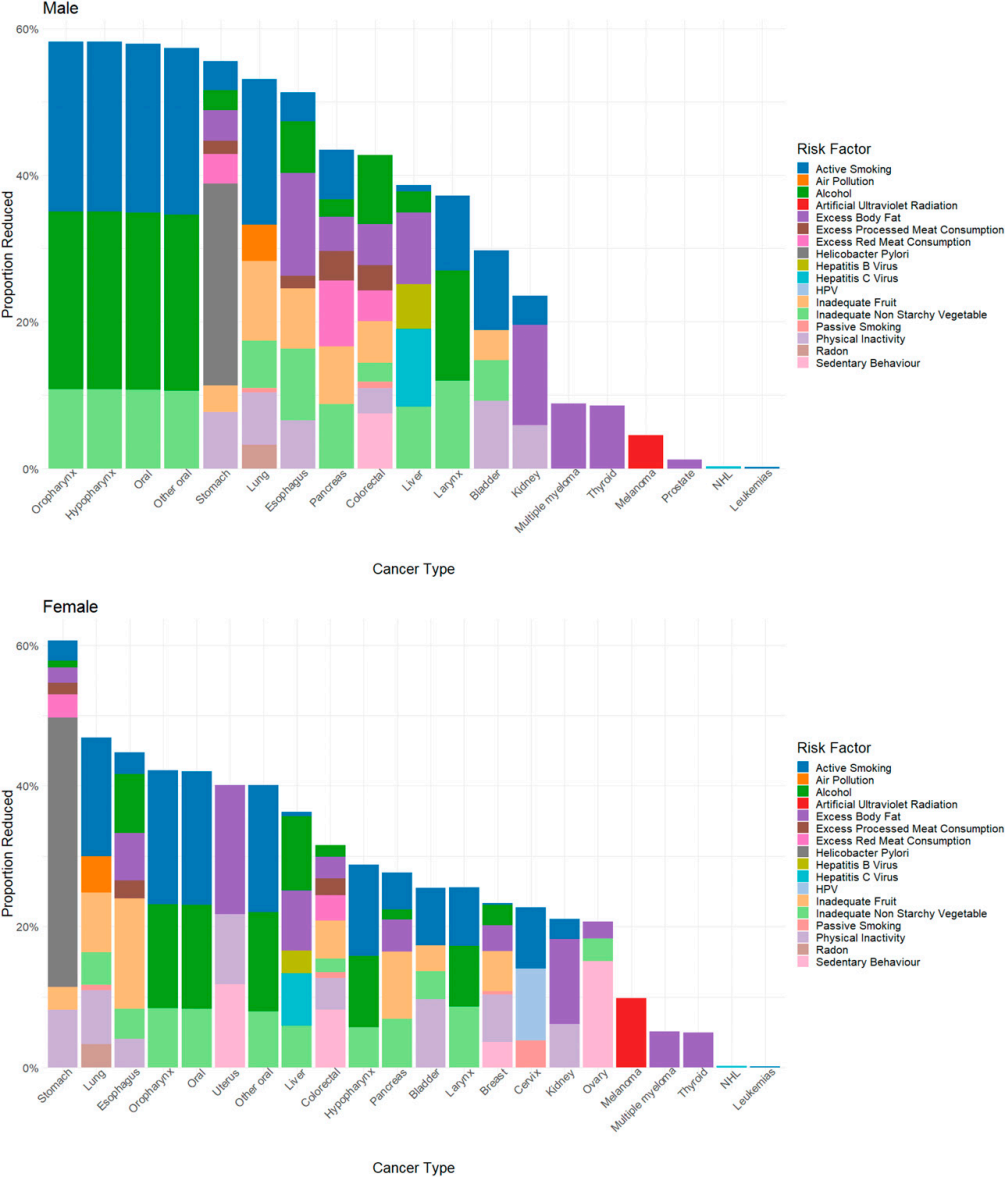
Preventable cancer deaths in the year 2050 across cancer sites. Each bar is divided by risk-factor intervention contribution.

Primary prevention through mitigation of the modifiable risk factors could prevent up to 22,173 cancer deaths among males (31.4%) and 17,865 cancer deaths among females (28.5%) in 2050 (Table 2). Figure 2 shows the number of preventable cancer deaths attributed to each of the risk factors by cancer site. Eliminating all active smoking accounted for the highest proportion of preventable deaths (7.6% for females, 9.5% for males), followed by eliminating inadequate fruit intake (5.7% for females and 6.1% for males) and eliminating physical inactivity (5.2% for females and 4.3% for males). The proportion of cancer deaths that could be prevented by mitigating each risk factor is shown in Supplemental Table 3.

**Table 2.** Percent of Prevented Deaths due to Primary, Secondary and Projected Prevented Deaths From Tertiary Prevention in Order to Reach a 50% Reduction in Cancer Deaths in 2050. Results are Shown for Males and Females.

Sex	All Cancer Deaths	Preventable Deaths due to Primary Prevention	Percent Impact of Primary Prevention (%)	Preventable Deaths due to Secondary Prevention	Percent Impact of Secondary Prevention (%)	Projected Preventable Deaths due to Tertiary Prevention	Projected Percent Impact of Tertiary Prevention (%)
Female	62,659	17,865	28.51	2318	3.70	11,153	17.80
Male	70,736	22,173	31.35	1344	1.90	11,884	16.80

Primary prevention is the result of maximally lowering exposure of cancer risk-factors. Secondary prevention is the result of screening programs for breast, lung, colorectal and cervical cancer. The remaining percentage to meet the Moonshot goal of 50% prevented cancer deaths could be accounted for through tertiary prevention (cancer treatment or diagnostic complications). These results show the preventable deaths in the scenario that primary prevention occurs first, then secondary, then the remaining gap is assigned for tertiary prevention.

**Figure 3.**
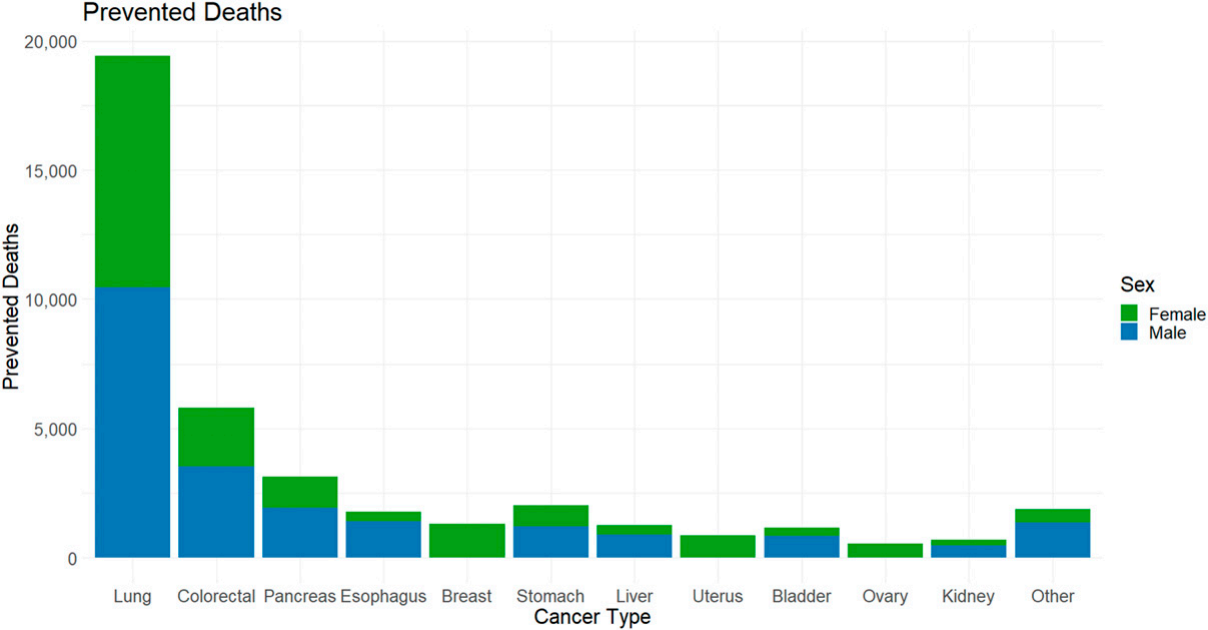
Preventable cancer deaths in the year 2050 across cancer sites. Each bar is divided by male and female preventable deaths. Other cancers include: Oral, oropharynx, hypopharynx, other oral, larynx, melanoma, cervical, brain, thyroid, Hodgkin’s lymphoma, non-Hodgkin’s Lymphoma, myeloma, leukemia, vulva, vaginal, anal, penile, prostate, and testicular cancer.

When stratified by cancer type, most preventable deaths were for lung cancer (male: 10,482; female: 8989), accounting for nearly half of all preventable deaths (Figure 3). In terms of the proportion of preventable deaths of each cancer type, males and females showed differing patterns (Figure 2). Among males, the highest proportions of preventable cancer deaths were seen for oral cancers (oropharynx, hypopharynx, oral, and other oral sites), which all had a proportional reduction of around 58%, followed by stomach (56%), lung (53%), and esophageal cancers (51%) (Figure 2). Among females, the highest proportion of preventable deaths were for stomach cancer (61%), followed by lung (47%) and esophageal (45%) cancers (Figure 2).

### Screening

The maximal effect of screening to reduce the mortality of lung, breast, colorectal and cervical cancer was simulated independent of any risk factor interventions. From the Screening-only model, breast cancer deaths could be reduced by 19.2%, colorectal cancer deaths by 21.9%, cervical cancer deaths by 36.4% and lung cancer deaths by 3.1% (Table 3). After accounting for risk factor intervention in the Combined model, the net effect of maximizing the effectiveness of screening programs was reductions of 14.7%, 13.7%, 28.1%, and 1.6% in cancer deaths for breast, colorectal, cervical and lung cancers, respectively, for a total of 3662 deaths (Table 3).

**Table 3.** The Proportion of Reduced Cancer Deaths due to Screening on Breast, Colorectal, Cervical and Lung Cancer in the Screening-Only and Combined Models.

	Proportion Reduced (Screening-Only) (%)	Proportion Reduced (Combined-Model Screening Impact) (%)
Breast	19.15	14.70
Colorectal	21.88	13.75
Cervical	^*^36.39	28.13
Lung	3.10	1.55

^*^10.83% of preventable deaths are associated with HPV vaccination.

Secondary prevention is the result of maximally saturating screening programs with at-risk individuals. These results show the affect screening programs would have with no changes in primary prevention.

### Combined Impact

**Figure 4.**
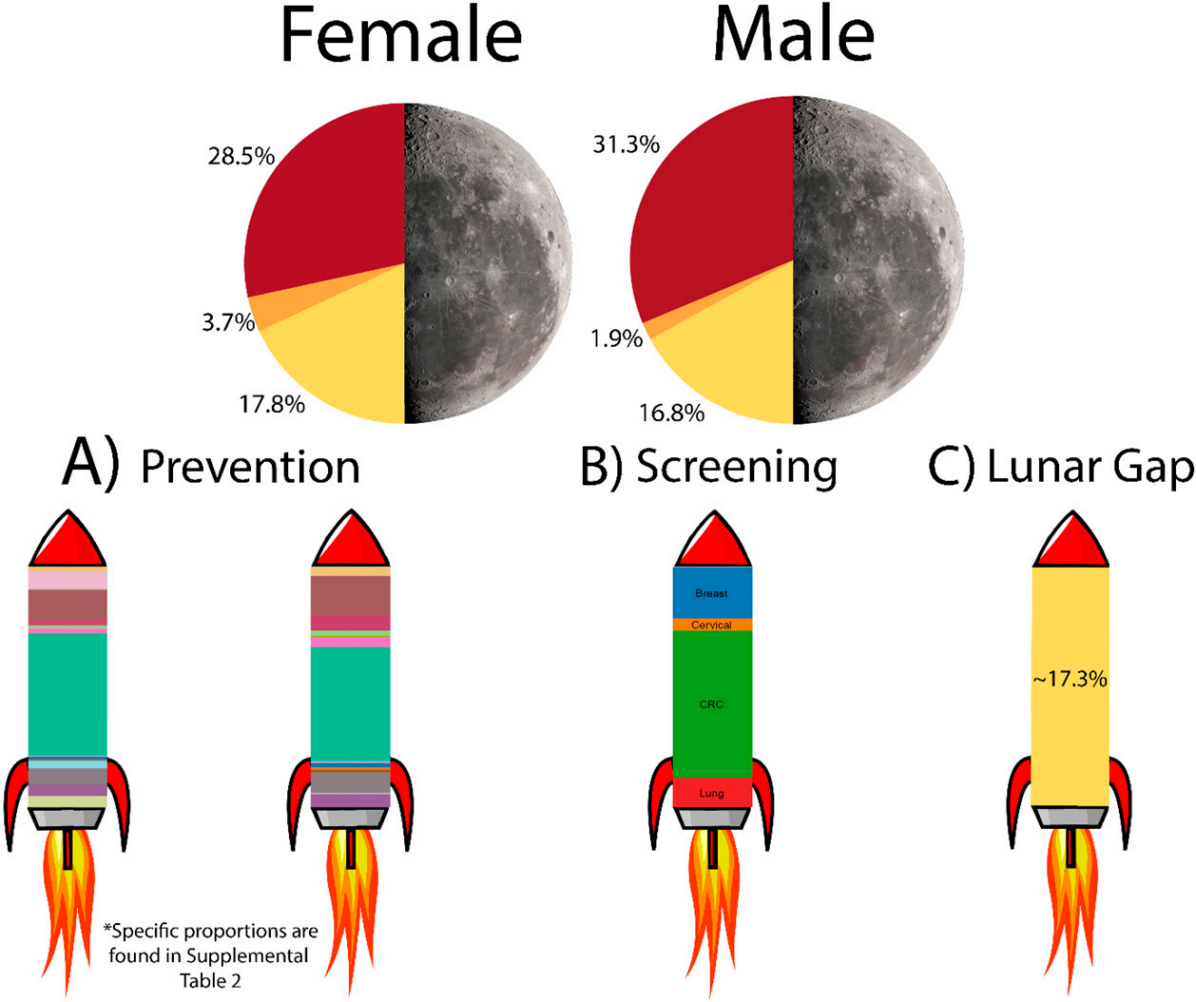
Potential impact of cancer prevention and screening for a Canadian Cancer Moonshot in 2050. The pie chart shows the maximal percentage of preventable deaths primary and secondary prevention can account for according to our models by the year 2050. The third slice depicts the minimal impact on preventable cancer deaths tertiary prevention required to reduce cancer deaths by half. Bar graph (A) shows preventable cancer deaths in the year 2050 for each cancer type. Bar graph (B) shows the ratio of preventable deaths due to cancer screening in 2050 for each cancer type screened. Bar graph (C) shows the gap in additional preventable deaths required for a 50% reduction in cancer deaths by the year 2050.

Combining primary and secondary prevention efforts, we projected a total of 20,186 (32%) and 23,501 (33%) preventable deaths for females and males, respectively, by the year 2050 (Supplemental Table 2). Prevented cancer deaths by respective cancer type split by gender is shown in Supplemental Table 4. Through primary and secondary prevention alone, we would be more than two-thirds of the way towards a 50% reduction in projected cancer deaths. This would leave a 16%-17% “lunar gap” to be bridged by tertiary prevention and future discoveries to reach the Moonshot initiative goal. The combined outcomes from the model are depicted in Figure 4.

## Discussion

This study provides estimates of the potential impact of a cancer moonshot program in Canada. Our results suggest that, under full population-level implementation of primary and secondary prevention efforts, a third of all cancer deaths could be prevented. However, in a real-world implementation, we would expect far fewer. These analyses suggest that a “Lunar Gap” of at least 16%-17% would exist between the current extent of knowledge on cancer prevention and a 50% reduction in projected cancer mortality. This gap highlights the necessity of advancements in new discoveries of risk factors, early detections, treatments, and clinical management. In reality, it is not feasible to achieve complete mitigation of most cancer risk factors and meet the 30% prevention target. For example, a 50% reduction in all modifiable risk factors, which is more reasonable yet still aspirational, would prevent about 15% of the cancer deaths, thus leading to a wider “Lunar Gap”.

The disparity between prevented mortality for primary and secondary prevention can be attributed to the number of screening programs available and screen detectable cancers. In the combined model, secondary prevention can only reduce mortality for cancers that have been detected, not for cancers that have been already prevented due to risk-factor reduction. The four screen detectable cancers (breast, lung, cervical and colorectal) accounted for 45% of cancer deaths in 2050. Prevented deaths varied between the four cancer types in the screening only model and the combined model. Cervical cancer was the only cancer type found to have more cancers prevented through screening only. While the existence of screening programs in Canada has reduced cancer mortality for breast, cervical and colorectal cancer, effectiveness of lung cancer screening in Canada is yet to be established.^20-22^ Current lung cancer screening programs have limited eligibility for high-risk smokers only. Over 70% of lung cancer cases are due to smoking which can be prevented through decreased risk-factor exposure.^23,24^

### Strengths & Limitations

This study generates projected data from OncoSim models and is bound by several assumptions around the uptake and adherence to screening programs and the risk exposure distributions to model risk factor reductions. While our assumptions may lead to optimistic results, the objective of this study is to stimulate discussion on the potential impact a Canadian Cancer Moonshot Initiative. The underlying trends of the prevalence of the lifestyle risk factors were projected from survey data up to 2011, which can be inaccurate if the risk factors are changing at faster or slower rates after 2011. For instance, our projected prevalence of obesity (25% in 2025) is lower than observed prevalence of 30% in 2022.^25^ OncoSim is updated on a regular basis, and we plan to update the projected risk factor prevalence in a future release. We only estimated the effect of HPV vaccination on cervical cancer, whereas HPV is known for causing several other types of oral and anogenital cancers.^26^ Therefore, we may have underestimated the total effect of HPV vaccination on cancer prevention. OncoSim presently does not include racial and ethnic diversity into model projections. There is evidence that links race and ethnicity to variable cancer outcomes.^27^ Canada’s high immigration rates could further impact our cancer mortality estimates next 25 years.

### Lunar Gap – What Else Do We Need?

With the maximal effects of primary and secondary cancer prevention, over 30% of projected cancer-related deaths can be prevented by 2050. This estimate highlights the significant advancements that has been made in cancer research however, to reach the 50% reduction goal, improvements need to be made across the cancer control spectrum to fill this gap.

### Filling the Gap with Primary Prevention

A significant portion of cancer deaths are preventable through risk-factor interventions. This estimate assumes that all individuals are reducing risk-factors to the theoretical minimum, which may be overly optimistic, as real-world adherence to risk reduction initiatives is typically much lower. Further research is needed to identify novel and emerging cancer risk-factors and to understand how best to implement prevention programs.

Research on preventive medicine is an emerging field for primary prevention of cancer. Chemopreventive agents such as aspirin has shown promising effects in reducing the incidence of colorectal cancer among the high-risk population.^28^ A few recent randomized controlled trials demonstrated that glucagon-like peptide-1 receptor agonists (GLP1RAs) in conjunction with diet and exercise are highly effective in treating obesity.^29,30^ On the population level, the use of GLP1RAs might prevent 1.3% of all cancers or 3.5% of obesity-related cancers by 2049.^31^

Education regarding cancer risk can be influential in motivating behavioral changes to reduce an individual’s exposure to risk-factors.^32^ A European study found that most people perceived genetics, alcohol, tobacco, and environmental pollution as the main sources of cancer.^32^ Less than 50% of people surveyed in this study were aware that unhealthy diets and UV radiation are risk factors of cancer.^32^ They also rated health education and promotional activities as unsatisfactory with over 60% of people not being informed about cancer prevention by their primary health care clinics.^32^ A recent study examined Canadians beliefs on true and false facts about cancer risk-factors.^33^ About 70% of facts were correctly identified, however Canadians in the study believed about 50% of the cancer risk-factor myths.^33^ Evaluating Canadians knowledge of cancer prevention can reveal weaknesses in our current system that can be improved.

### Filling the Gap with Secondary Prevention

To bridge the lunar gap, screening can be just as impactful as primary prevention strategies for reducing cancer mortality where screening programs exist. Increased exposure and promotion of screening programs can improve their participation. It is likely that in the coming decades additional screening modalities will be developed and implemented for cancers with existing screening programs as well as for new cancer sites. Multi-cancer early detection (MCED) tests have shown great promise as early cancer detection tools. Circulating tumor DNA and protein biomarker-based tests are the frontrunner methods for current clinical studies of MCED tests.^23,34^ MCED tests specificity has been reported to be 98% or more for all stages of cancer, however currently, sensitivity remains below 80% for cancers stage III and earlier.^34^ This is a challenge as MCED tests primarily benefit patients in the earliest stages of cancer compared to other cancer detection types.^34^ Decreasing false-positive rates and reducing patient anxiety from overdiagnosis and overtreatment are additional challenges currently being investigated to improve cancer detection approaches.^23,34^

### Tertiary Prevention – Improvements in Treatment and Clinical Management

Tertiary prevention refers to treatment and clinical management of the cancer once it has been diagnosed, in order to prevent recurrence, progression of disease, and death. Advancements in clinical management of cancers including the use of novel therapies has steadily improved survival and mortality for many common cancers. Analyses of population-level real-world data from non-small cell lung cancer patients in Alberta, Canada observed that molecular diagnostics and targeted treatments have had a meaningful impact on cancer survival and mortality at the population level.^35^ Additional discoveries will be needed to meet the Moonshot challenge. Tertiary prevention also aims to reduce cancer recurrence and development of new primary cancers. The risk of a second cancer among Canadians previously diagnosed with cancer is about 5 percent.^36^ Attention and research to mitigate the morbidity and mortality from second primary cancers is required for the increasing number of Canadians living with and beyond cancer.

## Conclusions

In this study, we projected cancer mortality in Canada until 2050 given current trends, estimated the maximal impact on reducing cancer mortality with reduction in known cancer risk factors and screening activities, and discussed how to achieve a 50% reduction in projected cancer mortality throughout the cancer prevention spectrum. While current knowledge and implementation of prevention and screening would have a considerable impact on reducing cancer mortality, additional efforts and investments are needed to for knowledge translation and for additional discoveries to achieve the aspirational “Moonshot” goal.

## Consideration of Sex and Gender

In this study, considerations for sex (biological) have been considered. The outcomes of female affecting cancers (breast, cervical) and male affecting cancers (testicular, prostate) have been estimated in this study. Cancer incidence and mortality estimates examined were separately and jointly account for differences. Breast and cervical cancer screening was only modeled for females. No gender (socio-cultural) considerations are applicable in this study as population-level cancer outcome data by gender are not presently available in Canada. Gender differences in cancer do exist (HPV screening, breast cancer mammography’s, etc.) however out study population was simulated and did not account for these differences.

## Contributions to Knowledge

What does this study add to existing knowledge?

• An estimation of cancer mortality and incidence by the year 2050.
• An estimate of preventable cancer deaths due to risk factor reduction and screening programs by the year 2050.
• The potential impact of implementing a Cancer Moonshot Initiative in Canada with the goal of reducing cancer mortality by 50% by the year 2050.
• How large is the gap between after accounting for projected cancer mortality reductions based on current trends and knowledge.

What are the key implications for public health interventions, practice, or policy?

• Current cancer mortality interventions can prevent around thirty percent of cancer deaths by the year 2050.
• Estimates found in this study highlight the substantial progress made to cancer outcomes.
• Advancements in primary, secondary, and tertiary prevention can also increase projected reductions in cancer mortality.
• The purpose of this study is to spark discussion on research to improve the future of cancer outcomes in Canada.

## Supplemental Material

Supplemental Material - Shooting for the Moon: Can We Cut Cancer Mortality in Canada By 50% By 2050? Supplemental Material for Shooting for the Moon: Can We Cut Cancer Mortality in Canada By 50% By 2050? by Keaton Banik, Yibing Ruan, Mariet M. Stephen, John M. Hutchinson, Chantelle Carbonell, Matthew T. Warkentin, Andrew Coldman, Rochelle Garner, Hawre Jalal, and Darren R. Brenner in Cancer Control
